# Plant Defense Responses Induced by Two Herbivores and Consequences for Whitefly *Bemisia tabaci*

**DOI:** 10.3389/fphys.2019.00346

**Published:** 2019-04-04

**Authors:** Dan Lin, Yonghua Xu, Huiming Wu, Xunyue Liu, Li Zhang, Jirui Wang, Qiong Rao

**Affiliations:** ^1^School of Agriculture and Food Science, Zhejiang A&F University, Hangzhou, China; ^2^Zhejiang Branch of National Pesticide R&D South Center, Zhejiang Chemical Industry Research Institute, Hangzhou, China

**Keywords:** *Bemisia tabaci*, *Phenacoccus solenopsis*, *Tetranychus cinnabarinus*, plant defense, jasmonic acid pathway, defensive enzyme, salicylic acid pathway

## Abstract

Diverse herbivores are known to induce various plant defenses. The plant defenses may detrimentally affect the performance and preference to subsequent herbivores on the same plant, such as affecting another insect’s feeding, settling, growth or oviposition. Here, we report two herbivores (mealybug *Phenacoccus solenopsis* and carmine spider mite *Tetranychus cinnabarinus*) which were used to pre-infest the cucumber to explore the impact on the plants and the later-colonizing species, whitefly *Bemisia tabaci*. The results showed that the whiteflies tended to select the treatments pre-infested by the mites, rather than the uninfected treatments. However, the result of treatments pre-infested by the mealybugs was opposite. Total number of eggs laid of whiteflies was related to their feeding preference. The results also showed that *T. cinnabarinus* were more likely to activate plant jasmonic acid (JA) regulated genes, while mealybugs were more likely to activate key genes regulated by salicylic acid (SA). The different plant defense activities on cucumbers may be one of the essential factors that affects the preference of *B. tabaci*. Moreover, the digestive enzymes and protective enzymes of the whitefly might play a substantial regulatory role in its settling and oviposition ability.

## Introduction

In nature, plants possess a considerable diversity of resistance strategies and produce complex chemical reactions after experiencing mechanical damage or attacks by herbivores (Green and Ryan, 1972). The signal transduction pathways related to plant defense includes the ethylene (ET) pathway, jasmonic acid (JA) pathway, and salicylic acid (SA) pathway. The JA and ET pathway are induced against necrotrophic pathogens, chewing herbivores and cell-content feeders (Thaler et al., 2012; Godinho et al., 2016). However, the SA signaling pathway is primarily induced by bio-trophic pathogens and piercing- sucking herbivores, resulting in minimal tissue damage (Arena et al., 2016). Simultaneously, insects adapt to plant defense strategies by evolving their feeding patterns and feeding behavior (Hogenhout and Bos, 2011). In some cases, the herbivores may induce the accumulation of SA by attacking host plants and utilize this accumulation to inhibit JA-mediated defenses. That is, interference between the plant defense pathways may occur (Zhang et al., 2011; Zhang X. et al., 2015). The interaction between separate signaling pathways in host plants carries out an irreplaceable role in regulating plant defense and preventing further invasions of pests. Between them, the JA pathway and SA pathway consistently showed mutual inhibition (Pieterse et al., 2012; Thaler et al., 2012; Glas et al., 2014). For example, several genes (like *LOX* and *OPR3*) regulated by the JA pathway can be restrained by the SA signaling pathway in Arabidopsis, suggesting that SA intensely antagonizes the JA signaling pathway (Guleria et al., 2005). Furthermore, ET is a critical modulator of SA-JA cross-talk, which equally participates in the regulation of a key gene (*NPR1*) in the SA pathway. Thereby, it regulates the inhibition of the JA pathway. As follows, the presence of the ET pathway is equally involved in the complex interaction between the SA pathway and the JA pathway. This intervention is mediated through the regulation of the NPR1 protein (Guleria et al., 2005; Thaler et al., 2012). Collectively, the cross-talk between JA, ET and SA signaling is ultimately to balance plant-defense strategies in response to multiple attackers (Guleria et al., 2005).

The complicated induced plant defenses may affect the performance and preference of subsequent herbivores, by affecting the feeding, settling, growth, or oviposition ability of another insect (Karban and Carey, 1984; Leitner et al., 2005). For instance, the plant may become more sensitive to another herbivore after previous attacks by other herbivores. Meanwhile, the phloem-feeding insects were susceptible to the change of nutrient substance and resistant material in the phloem sap (Denno and Roderick, 1992). A substantial number of secondary metabolites in plants induced by pests are extremely detrimental to the growth performance of insects, like terpenoids, phenolic compounds, and alkaloids (Chen et al., 2015). The resistance of insects to the secondary metabolites is due to various biochemical and physiological characteristics of the midgut. The midgut of insects can secrete various digestive enzymes (e.g., proteases, lipases, amylases, sucrases, trehalases) and defense enzymes (superoxide dismutase, catalase, and peroxidase), which occupy a crucial role for insects to combat plant defenses.

The whitefly *Bemisia tabaci* (Gennadius) is a phloem-feeding pest that causes extensive damage (De Barro et al., 2011; Liu et al., 2012). Due to the wide host range, intense competition exists between the invasive species *B. tabaci* and other local pests (De Barro et al., 2011; Rao et al., 2011). The carmine spider mite *Tetranychus cinnabarinus* (Boisduval) is a polyphagous pest that attacks crops, vegetables, and flowers. In particular, the economic yield of cucumbers decreases significantly after heavy spider mite infestations (Sertkaya et al., 2010; Park and Lee, 2016). The spider mite remains a cell-content feeder, which can puncture the host plant leaf epidermis cells and consume the contents of the mesophyll cells. Typically, the mite induces defenses of both the SA and JA pathways (Ament et al., 2004). Moreover, *T. evansi* down-regulates tomato defensive compounds, and this is correlated with a significantly more excellent performance of herbivores on plants pre-infested by mites of this species (Kant et al., 2008; Alba et al., 2015; Godinho et al., 2016). The mealybug *Phenacoccus solenopsis* (Tinsley) represent an aggressively invasive species on agricultural and ornamental plants in China. It is an obligate phloem-feeding pest that possesses a specialized stylet and causes minimal damage as it obtains nutrition from plants through the vascular tissue (Will et al., 2013; Huang and Zhang, 2016). All three pests have broad host distributions, and can damage the same crops in the field. Previous studies have shown diverse pests that eat plants often activate different signaling pathways (Zhang et al., 2011). Here we pre-infested cucumbers with *T. cinnabarinus* and *P. solenopsis*, insects with different foraging patterns, to explore the impact on *B. tabaci*.

## Materials and Methods

### Plant and Insects

#### Plant

Cucumber, *Cucumis sativus* L. (Jinongjiejiegua, Tianjin, China), was the host plant for this experiment. The plants used in the tests were approximately 30 cm in height and had 4–6 true leaves.

#### Insects

The spider mites, *T. cinnabarinus*, were maintained on broad bean plants (*Vicia faba*). The mealybugs, *P. solenopsis*, were cultured on potato plants (*Solanum tuberosum*). The whiteflies, *B. tabaci* (MED), were cultured on cucumber plants (*Cucumis sativus*).

The experiments were conducted at 25 ± 2°C, 65 ± 10% RH and a photoperiod of 16 h:8 h (L:D) with artificial lighting in a greenhouse.

### The Settling and Oviposition of Whitefly

The leaf disks were pre-infested by five 3rd-instar female mealybugs or five red mite females for 12 h. Then, five couples of new emerged whiteflies were placed at the middle of the plastic pipe and allowed to fly to either side. The positions of the two leaf disks were alternated among replicates. Each experiment was replicated 15–30 times. In the experiment, the location of *B. tabaci* was counted every 12 h. After 72 h post inoculation (hpi), the number of laid eggs deposited on each leaf disk was recorded. In total, there were 15–30 replicates in each treatment. The ratios of the whitefly number of landing on the plant to the total number of releasing in the test were subjected to a *t*-test (SPSS version 18.0).

### Enzymatic Assay of Defensive Enzymes in Host Plants

The activity of defensive enzymes in plants was tested as follows: (1) T(+), each plant pre-infested by 25 *T. cinnabarinus* females for 12 h and continue feed for 3 days; (2) P(+), each plant pre-infested by 25 *P. solenopsis* for 12 h and continue feed for 3 days; (3) T(+)+B, after pre-infested for 12 h, the *T. cinnabarinus* were kept and *B. tabaci* was infested for 3 days; (4) P(+)+B, after pre-infested for 12 h, the *P. solenopsis* were kept and *B. tabaci* was infested for 3 days; (5) CK, the plants were uninfected but were kept for the same time and were used as controls.

Enzymatic assay of PAL was determined according to the procedure of Burrell and Rees (1974). The enzymatic assay of LOX is slightly modified according to the method described by Surrey (1964). This experiment contains 5–8 biological experiments with three technical repetitions.

### Quantitative Real-Time PCR

We quantified the transcript levels of several genes in the leaves treated. Leaves of the treated and untreated plants were collected, and total RNA was extracted consuming the RNAiso Plus reagent (TaKaRa, Dalian, China). The cDNA was synthesized using the PrimeScript^®^RT Reagent Kit with gDNA Eraser (TaKaRa, Dalian, China). For the relative quantification of gene expression, the comparative *CT* method (Livak and Schmittgen, 2012) was used with BIO-RAD CFX96 real-time PCR system (Bio-Rad, United States). The amount of the target was normalized to the endogenous reference gene *TUA*. The specific primers of all aimed genes for qRT-PCR were showed in Table 1. For qRT-PCR, three biological replicates were carried out, and triplicate quantitative assays for each replicate were performed.

**Table 1:** The specific primers for qRT-PCR.

Gene	Accession No.	Forward primer	Reverse primer
*TUA*	XM011654602	AGCGTTTGTCTGTTGACTATGGA	TGCAGATATCATAAATGGCTTCGT
*PAL*	XM011660164	TTGGTGGTGAAACTCTTA	AACTATCGGTCCCTTTAT
*OPR3*	XM004143979	ACTTCCATCTCCGCCACT	TGAACAGCATCCACCACT
*LOX2*	NM001305766	ACCGAAAGTGACCAACGC	CCACGAATGATTAGAGGG
*PR1*	GQ229574	TGCTCAACAATATGCGAACC	TCATCCACCCACAACTGAAC
*PR5*	XM011651301	CTATCGGCTCAAGCAGTG	ACGGGCAGATTCCATTCA
*ETR*	NM001280633	GCCATTGTTGCAAAAGCAGA	GCCAAAGACCACTGCCAC

### The Digestive Enzymes, Detoxification Enzymes, and Protective Enzymes in Whiteflies

Here, the detoxification, digestive and protective enzymes in whiteflies were also tested to reveal the effect of induced plant response. The different treatments were as follows: (1) T(+)+B (described above); (2) P(+)+B (described above); (3) Whitefly (the plants that were uninfected but were kept for the same time, and then 25 couples of whiteflies were allowed to feed on the plants as a control). The whiteflies were collected at the end of the experiment and each treatment was repeated 8–10 times.

For the determination of trehalose and sucrase activity, the dinitrosalicylic acid method was adopted (Zhang et al., 2014). For the determination of superoxide dismutase (SOD) activity, the pyrogallol auto-oxidation method was used (Xu et al., 2006). The molybdate colorimetric method was used for determination of catalase (CAT) activity (Cheng and Meng, 1994). The glutathione S-transferase (GST) activity was measured according to Habig and Jakoby (1981). The carboxylesterase (CarE) activities were performed with slight modifications according to Stumpf and Nauen (2002).

## Results

### Effects of Mealybug or Spider Mite Infestation on *B. tabaci* Response

**FIGURE 1:**
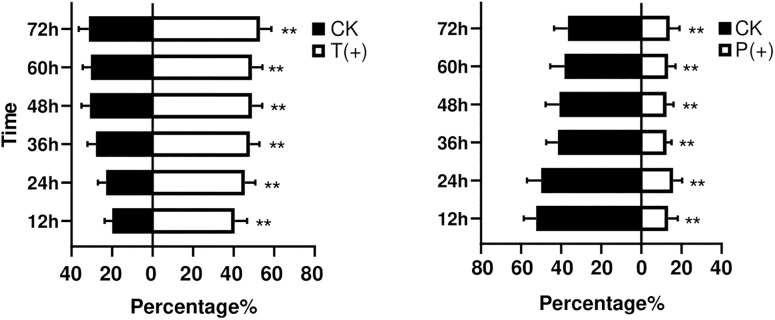
Mean percentages (± SE) of *B. tabaci* adults selection over different observation times. (^∗∗^ Independent *t*-tests: *P* < 0.01).

**FIGURE 2:**
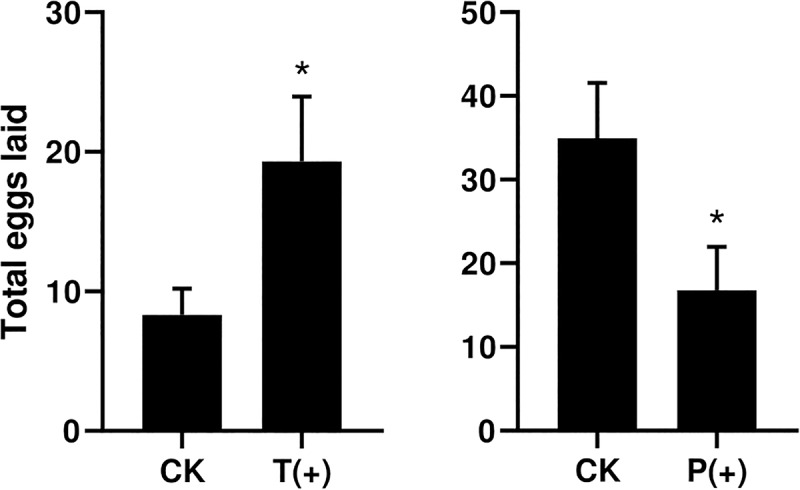
Total number of eggs per dish of *B. tabaci* fed for 3 days in selective bioassays. (^∗^ Independent *t*-tests: *P* < 0.05).

The percentages of *B. tabaci* that settled on the treatments with *T. cinnabarinus* were significantly more than those on the control treatments without *T. cinnabarinus* (12 h, *P* = 0.008; 24 h, *P* = 0.002; 36 h, *P* = 0.003; 48 h, *P* = 0.009; 60 h, *P* = 0.007; 72 h, *P* = 0.007) (Figure 1). In contrast, less whiteflies settled on the plants infested with *P. solenopsis* (12 h, *P* = 0; 24 h, *P* = 0.001; 36 h, *P* = 0; 48 h, *P* = 0.001; 60 h, *P* = 0.005; 72 h, *P* = 0.014) (Figure 1). In all, *B. tabaci* were more likely to choose the leaf disk with *T. cinnabarinus* treatment than with *P. solenopsis* treatment. After 3 days, the total laid eggs were significantly higher in the plants with *T. cinnabarinus* than those on the leaf disk with no treatment (Figure 2). In contrast, the total eggs were significantly lower in the plants with *P. solenopsis* than control (Figure 2).

### The Digestive Enzymes, Detoxification Enzymes, and Protective Enzymes in Whiteflies

According to Table 2, we found the trehalose, SOD and CAT activities in *B. tabaci* from cucumbers infected by mites were higher than those of whiteflies from the uninfected cucumber (*P* < 0.05). However, the sucrase and GST activities were significantly lower than those of whiteflies reared in cucumbers infected by mites (*P* < 0.05). The CarE activity of *B. tabaci* from cucumber fed on by mealybugs was higher than that of whiteflies from cucumbers infected by mites and the control. Similarly, the sucrase activity in whiteflies was significantly lower from cucumber infected by mealybugs than that of whiteflies in the control (*P* < 0.05).

**Table 2:** The digestive enzymes, detoxification enzymes and protective enzymes in whiteflies.

Treatments	Whitefly	T(+)+B	P(+)+B
Trehalose activities (mmol/min⋅mgpr)	0.29 ± 0.05*a*	0.58 ± 0.08*a*	0.49 ± 0.05*ab*
Sucrase activities (mmol/min⋅mgpr)	0.71 ± 0.14*a*	0.21 ± 0.10*b*	0.03 ± .01*b*
Carboxylesterase activities (μmol/min⋅mgpr)	2.82 ± 0.40*b*	3.47 ± 0.23*b*	6.20 ± 1.47*a*
glutathione S-transferase activities (ΔOD340/min⋅mgpr)	3.38 ± 0.10*a*	1.40 ± 0.20*b*	3.09 ± 0.14*a*
Superoxide Dismutase (SOD) activities (U/min⋅mgpr)	0.24 ± 0.01*b*	0.78 ± 0.22*a*	0.28 ± 0.05*b*
Catalase activities (μmol/min⋅mgpr)	0.16 ± 0.02*b*	0.40 ± 0.03*a*	0.22 ± 0.03*b*

*Paired means (± SE) with different letters above bars indicate significant differences in the quantities between control and treatment (ANOVA, P < 0.05).*

### Estimation of Phenylalanine Ammonialyase and Lipoxygenase Activity in Plants

**FIGURE 3:**
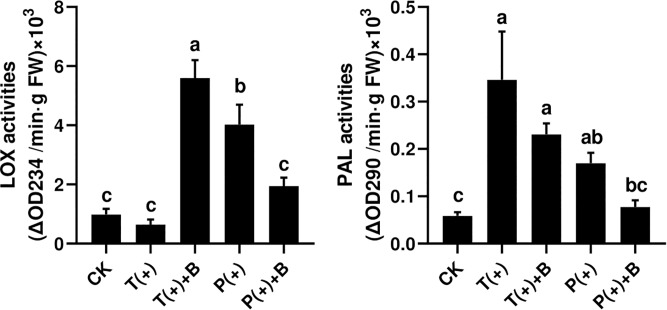
The activity of two defense enzymes in each treatment on cucumber. Different letters above bars indicate significant differences in the quantities between control and treatment (ANOVA, *P* < 0.05).

The activity of PAL but not LOX was significantly increased through the induced response in cucumbers fed on by spider mites after 3 days. However, the activities of LOX and PAL in cucumbers were significantly increased by feeding mealybugs. The activities of LOX and PAL in cucumbers were both significantly increased after the whiteflies coexisted with mites for 3 days. Nevertheless, the activities of LOX and PAL in cucumbers were not significantly increased after the whiteflies coexisted with mites for 3 days (Figure 3).

### Changes in Gene Expression

**FIGURE 4:**
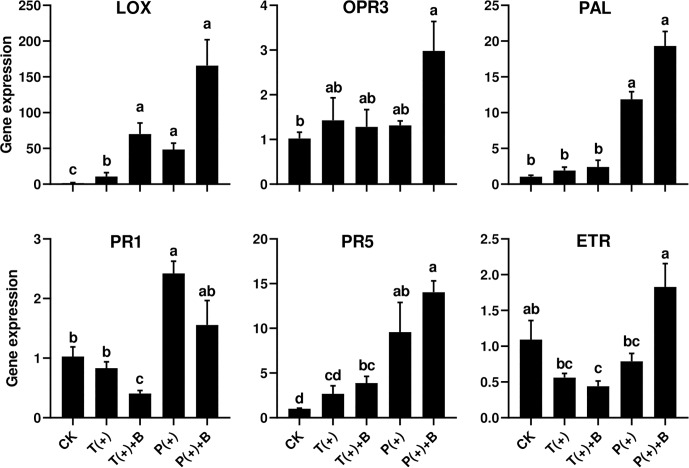
Effect of pre-infestation of *T. cinnabarinus* and *P. solenopsis* on the expression levels of *LOX*, *OPR3*, *PAL*, *PR1*, *PR5*, and *ETR* genes in leaves of different combinations. Different letters above bars indicate significant differences in the quantities between control and treatment (ANOVA, *P* < 0.05).

To assess cucumber response to five different combinations of the insect feeding treatments, time course experiments were conducted in which transcript levels of one ET-dependent gene (*ETR*), two JA-dependent genes (*LOX2* and *OPR3*) and SA-dependent genes (*PAL*, *PR-1*, and *PR-5*) were monitored (Figure 4).

Ethylene receptor gene (*ETR*) is a marker gene of ET signal pathways (Shoresh et al., 2005). The relative expression levels of *ETR* did not significantly change in the cucumber leaves infested with *T. cinnabarinus* only or *P. solenopsis* only after 3 days. However, the transcript levels of *ETR* in the cucumber leaves induced by *T. cinnabarinus* were repressed in leaves infested with both *T. cinnabarinus* and *B. tabaci* compared to undamaged leaves (*P* < 0.05) (Figure 4).

Lipoxygenase (*LOX*) is typically a key enzyme in the octadecanoid pathway of the JA biosynthesis (Hu et al., 2009). All treatments apparently upregulated the transcript levels of *LOX* compared to undamaged leaves. However, the level of *LOX* induction in leaves infested with *T. cinnabarinus* was significantly lower than other treatments (Figure 4). The oxophytodienoate reductase (*OPR3*) gene is one marker of the JA upstream genes (Ramírez et al., 2012). The level of the *OPR3* gene infested with either *P. solenopsis* or *B. tabaci* was significantly higher than other treatments (*P* < 0.05) (Figure 4).

*PR-1* and *PR-5* are known as two pathogenesis-related genes, which are mainly regulated by the SA signaling pathway (Zhang P.J. et al., 2015). Phenylalanine ammonia lyase (*PAL*), a rate-limiting enzyme of the phenylpropanoid pathway, is the key enzyme in the biosynthetic pathway of SA and plays an important role in plant development and defense (Hu et al., 2009; Wang et al., 2013; Zhang et al., 2016). The abundance of *PAL*, *PR1*, and *PR5* transcripts was significantly increased in the cucumber leaves infested with *P. solenopsis* only after 3 days, but not in the leaves infested with *T. cinnabarinus* only (*P* < 0.05; Figure 4). In addition, the leaves infested with *P. solenopsis* and *B. tabaci* showed a marginally significant increase of the expression of *PAL* and *PR5* in comparison with undamaged leaves. However, the leaves infested with *P. solenopsis* and *B. tabaci* witnessed a significant reduction of the expression of *PR1* compared to undamaged leaves (Figure 4).

## Discussion

From the perspective of temporal and spatial interactions, intraspecific insect interactions affect the population performance, especially because herbivores may affect the performance of subsequent herbivores on the same plant (Green and Ryan, 1972; Hunter and Price, 1992; Messina et al., 2002; Beale et al., 2006; Xue et al., 2010; Tan and Liu, 2014). Our result showed whiteflies were extremely sensitive to pre-infestation with mealybugs, though there were few previous reports of mealybugs. In contrast, the plants pre-infested with *T. cinnabarinus* attracted more whiteflies than the plants with no mites. This may depend on the combination of the activation of defensive proteins and volatile organic compounds (Smith and Boyko, 2007; Howe and Jander, 2008), as well as the species of pests and feeding sequences of herbivore insects (Inbar and Gerling, 2008; Erb et al., 2011). Plants known to be impacted by herbivores are capable of producing a series of volatile substances that trigger an indirect plant defense. How plants respond to infestation by multiple herbivores, particularly if these belong to different feeding guilds, is important. The pattern of the plant defense is crucially important for regulating the behavior of herbivorous pests (Walling, 2000). Transcriptomic evidence has demonstrated that chewing insects activate the JA pathway, whereas sucking pests usually elicit the SA pathway. Next, a cell-content feeder may induce defenses of both the SA and JA pathways (Zhang et al., 2009). In fact, inconsistencies in induced plant defense depend on the species of the pest or the host plant species (Walling, 2000; Diezel et al., 2009). The induced defense pathway is always considered to promote the production of secondary metabolites and is very unfavorable to insects (e.g., affecting pest feeding and digestion) (Walling, 2000; Jormalainen et al., 2001). In addition, the contents of the SA, total phenolic, and tannins were negatively correlated to the fecundity of *B. tabaci*. Simultaneously, the increase of plant secondary metabolites and the lack of nutrients may affect the feeding preference of *B. tabaci*.

Numerous investigations indicate that sucking insects activate the SA signaling defenses and inhibit the JA pathway more easily (Kempema et al., 2007; Poelman et al., 2012). For example, *B. tabaci* activate SA signaling and inhibit JA-dependent responses (Kempema et al., 2007; Zarate et al., 2007; Zhang et al., 2013a). *P. solenopsis* can limit the expression of JA-depended genes and defensive substances to enhance its nymph adaptive ability on cotton. Specifically, mealybugs could induce the expression of JA-related genes but reduce the production of JA-regulated defensive substances, like gossypol on cotton (Zhang et al., 2011). Due to the particular interaction between JA-SA, mealybugs can enhance the host adaptability of its nymphs (Zhang X. et al., 2015), but not for the performance of *B. tabaci*. Here, the result showed expression level of *LOX* was significantly higher in *P. solenopsis*- infested plants compared to undamaged plants. However, *T. cinnabarinus* had less ability to induce *LOX* genes in comparison to *P. solenopsis*. *T. cinnabarinus* remain a typical cell-content feeder that may activate JA and SA pathways. Previous research confirmed that insects and mites could suppress the JA pathway to enhance their performance on the same plant, but maybe not for another species (Sarmento et al., 2011; Glas et al., 2014). Here, *P. solenopsis* significantly upregulated the expression level of the *PR* genes and *PAL* gene, a downstream gene regulated by SA. However, *T. cinnabarinus* appeared to be following the reverse strategy. Although it has previously been reported, that mealybugs and aphids can upregulate PR genes after feeding on hosts (Zhang X. et al., 2015). The direct and substantial roles of PR proteins in defense against various threats (including insects) show diversified mechanisms (Ebrahim et al., 2011), which are not very clear against the phloem-feeding insects. In contrast, JA-regulated genes may perform a crucial role in preventing pest invasions (Rahbé and Febvay, 1993). In our study, a significantly higher level of the *LOX* gene was observed on leaves pre-infested with mites or mealybug after 3 days. The results suggested the effectiveness of plant defenses elicited by upregulating the level of *LOX* gene against *P. solenopsis*’ invasion. Taken together, it is important to emphasize that JA/ET-induced defenses carry out an important role in the successful defense against the phloem-feeding pest, like *B. tabaci*. The *P. solenopsis* maintains a ‘stealthy feeding strategy’ that avoids massive cell damage, which may produce insufficient JA levels for gene induction (De Vos et al., 2006). There is antagonism between SA and JA, and the SA signaling pathway may inhibit the JA signaling pathway (Doares et al., 1995).

Previous studies have revealed pests always accumulate SA at the site of oviposition, which may interfere with the accumulation of JA (Bruessow et al., 2010). Therefore, the high content of PR protein not only does not adversely affect *B. tabaci*, but rather is conducive to reproduction and development (McKenzie et al., 2002). In addition, the leaves infested with *P. solenopsis* and *B. tabaci* showed a marginally significant increase of the expression of *PAL* and *PR5* by comparison with the undamaged leaves. However, the leaves infested with *T. cinnabarinus* and *B. tabaci* witnessed a significant reduction in the expression of *PR1* compared to undamaged leaves. Thus, the high expression of SA regulatory genes did not favor the selection and oviposition of *B. tabaci*. Another study confirmed that whitefly pre-infestation of the tomato reduced the fecundity of subsequent whiteflies (Cui et al., 2012). Moreover, JA-mediated defenses may in addition cause an effect on egg development (Bruinsma et al., 2007). This suggests that inhibition of plant defense is of importance to phloem feeders, allowing them to effectively utilize the host.

The cross-talk between JA-SA has been demonstrated adequately in the past. Studies have speculated insects can take advantage of this cross-talk to address plant defenses to enhance host adaptability (Zhang et al., 2013b). Like JA, it is equally common for ET signals in plants to be activated by insects. However, ET is not so much a direct stimulus as an indirect regulator, that rarely directly adjust the corresponding plant defense (Dahl and Baldwin, 2007; Diezel et al., 2009). In addition, the ET pathway may equally affect genes regulated by JA in numerous plants (Zhu-Salzman et al., 1998; Royo et al., 1999; Stotz et al., 2000; Winz and Baldwin, 2001). Moreover, the interference between plant hormones allows plants to minimize their own cost of capacity, thereby developing a flexible signaling pathway that is more effective against intruders (Zhang et al., 2009). The ability of plants to regulate signaling pathways may, in turn, provide benefits to other invasive pests (Sarmento et al., 2011; Glas et al., 2014). Comparing to the leaves infested with *T. cinnabarinus* only, *B. tabaci* caused a marginally significant reduction of the level of *ETR* and *PR1*. However, it facilitated the expression level of *LOX*, a JA-regulated gene.

Insects equally possess a range of defense strategies to adapt to harsh environments, including the adaptations of detoxifying enzymes, digestive enzymes and protective enzymes (Ahmad et al., 1986; Jongsma et al., 1995; Pearse et al., 2013). *B. tabaci*, as a sucking pest, will inevitably exchange material with the plant phloem. It is properly known that plants fed by pests can induce the production of secondary metabolites. These substances can stimulate the protective enzymes and detoxification enzymes of *B. tabaci*, affect the midgut digestive enzymes, and thus their feeding and digestion and absorption capacity (Green and Ryan, 1972; Ahmad et al., 1986). The difference in detoxifying enzymes frequently affects the interspecific competition between *B. tabaci* and other populations (Julian et al., 2003; Liang et al., 2007; Liu et al., 2008). In this study, the activity of GST in whiteflies was significantly inhibited after feeding by mites, and the activity of CarE increased significantly after co-existence of whiteflies and mealybugs. Previous studies have shown CarE is important for insect resistance and can detoxify a variety of environmental toxicants. Therefore, this study demonstrated that the increase in CarE activity of *B. tabaci* may be one of the adaptation measures for it against adverse environmental conditions.

Insects advocate broader tolerance to secondary metabolites, and this tolerance is determined by a variety of biochemical and physiological characteristics of the midgut. As one of the most key components of plant phloem, sucrose can supply energy for insects. Not only that, it can maintain osmotic pressure balance for sucking insects to ensure insects survivein a high osmotic pressure environment (Becker et al., 1996). Trehalose is known as the sugar of life. Its vital function is to synthesize chitinase and participate in the metabolism of insects, therefore providing energy for insect host positioning, courtship, mating, resisting adversity, spawning and other activities (Tang et al., 2012; Qin et al., 2015). Therefore, host adaptability may be reflected by the activity of trehalase and invertase in insects. *B. tabaci* may respond to the unfavorable situation by increasing trehalase activity. However, the activity of trehalase in whiteflies was inhibited on the plants infected by mealybugs, which may affect the behaviors of *B. tabaci*, like the oviposition behavior. It was only significantly increased after the pre-infestation of mites. Therefore, it was adequately demonstrated that the presence of mealybugs may cause an undesirable effect on the performance of *B. tabaci*, like the abilities of digestion and feeding. In addition, trehalase is also involved in feeding regulation and provides energy for insect flight behavior, which may be directly related to the tendency of *B. tabaci* in the field to select a host (Shi et al., 2016).

SOD can scavenge superoxide anion radicals and prevent damage to the body, but the generated hydrogen peroxide is still harmful to the insect midgut, however, CAT and POD in the body will immediately dispose of it (Tang et al., 2016). The protective enzyme activities of *B. tabaci* increased significantly after coexisting with *T. cinnabarinus*. However, this phenomenon does not occur when coexisting with mealybugs.

Because of the broad distribution and overlapping of hosts, all three pests in this study are extremely likely to erupt simultaneously on the consenting host plants in the field. Therefore, exploring the differences in plant defenses induced by different pests helps to reveal an understanding of the rules and mechanisms of pest invasion in the field. Despite previous reports of competition between mealybugs and whiteflies or spider mites and whiteflies, there has been increasing interest in the interspecies relationships between sap-feeding insects and the interspecific effects between mites and sucking insects (Godinho et al., 2016). In this study, *T. cinnabarinus* was more likely to activate plant genes regulated by JA, while mealybugs were more likely to activate key genes regulated by SA. Either may be one of the chief factors that affect the resistance of host plants to *B. tabaci*. Moreover, *B. tabaci* was more adaptable to plants coexisting with *T. cinnabarinus*, which may be attributed to the increase in digestive enzyme activity and protective enzyme activity. This result is of great significance to the layout of field crops and pest control or prevention. It additionally provides a firm basis for exploring the functions of the detoxification enzymes GST, trehalase and the protective enzyme systems in the anti-defense of *B. tabaci*. Through the further study of plant defense mechanisms, it is of great significance to understand the direct interaction of pests as well as the monitoring and control of field pests. These will support us to take effect early warning measures as early as possible and use natural laws to prevent and control natural disasters.

## Author Contributions

QR and HW conceived and designed the experiments. DL, YX, XL, LZ, and JW performed the experiments. DL and QR analyzed the data. DL and QR drafted the manuscript. All authors read and approved the final manuscript.

## Conflict of Interest Statement

YX was employed by company Zhejiang Chemical Industry Research Institute. The remaining authors declare that the research was conducted in the absence of any commercial or financial relationships that could be construed as a potential conflict of interest.
